# Open-Label Prospective Randomized Comparative Study of the Efficacy and Safety of Gentamicin in Comparison to Other Antibiotics in the Management of Acute Appendicitis in Surgically Treated Patients

**DOI:** 10.3390/antibiotics15040395

**Published:** 2026-04-13

**Authors:** Nika Obolnar, Žan Čebron, Gregor Norčič, Darko Černe, Aleš Jerin, Urška Čegovnik Primožič, Gaj Vidmar, Tadeja Pintar Kaliterna, Bojana Beović

**Affiliations:** 1 University Rehabilitation Institute, 1000 Ljubljana, Slovenia; 2 Department of Abdominal Surgery, University Medical Centre Ljubljana, 1000 Ljubljana, Slovenia; 3 Faculty of Medicine, University of Ljubljana, 1000 Ljubljana, Slovenia; 4 Institute of Clinical Chemistry and Biochemistry, University Medical Centre Ljubljana, 1000 Ljubljana, Slovenia; 5 Faculty of Mathematics, Natural Sciences and Informatics, University of Primorska, 6000 Koper, Slovenia; 6 Department of Infectious Diseases, University Medical Centre Ljubljana, 1000 Ljubljana, Slovenia

**Keywords:** appendicitis, intra-abdominal infection, antibiotics, aminoglycosides, nephrotoxicity

## Abstract

Background: Antimicrobial resistance coupled with the lack of new antibiotics calls for the responsible use of antibiotics, including old antimicrobials. Aminoglycosides are effective against bacteria in acute appendicitis, a common intra-abdominal infection. Their use has been discouraged recently, but their place in therapy is based on studies performed in the era of lower resistance rates, and with multiple dosing regimens. Methods: In a prospective randomized open-label study, we compared the efficacy and safety of gentamicin in one daily dose and metronidazole (GTM+MZ) to ertapenem (ETP) and to cefuroxime with metronidazole (CXM+MZ) in adult patients surgically treated for acute appendicitis. Efficacy was assessed via the duration of antibiotic treatment and hospital stay, c-reactive protein (CRP) dynamics, and post-operative complications. Nephrotoxicity was assessed with urine biomarkers. Statistical analysis comprised mixed-model analysis of variance (ANOVA) with the missing-data-imputation method and linear mixed model (LMM). Results: One hundred-and-sixty-six patients were included in this study. There were no significant differences among the three groups in the durations of treatment and lengths of stay (*p* = 0.093, *p* = 0.222). CRP level was the lowest (*p* = 0.003) in the ETP group. There were five complications during hospitalization, with two of them classified as infectious. Both occurred in the GTM+MZ group; however, the difference was not statistically significant (*p* = 0.330). No difference was found in complications in the month following the operation *(p* = 0.763). Biomarkers indicating kidney injury showed the same trend in all three groups. Conclusions: Our results suggest the use of once-daily dose of gentamicin following an appendectomy for acute appendicitis. Gentamicin may be used to decrease selective pressure of other antimicrobials.

## 1. Introduction

Acute appendicitis is the most common surgical condition in abdominal surgery that, in the majority cases, requires an urgent operation. The incidence of acute appendicitis is 7–10%. The risk of developing acute appendicitis is 8.6% in men and 6.7% in women. Management depends on disease grade. The American Association for the Surgery of Trauma (AAST) divided appendicitis into five grades, where grade I (intact appendix) could be managed with non-operative therapy only [1]. Antibiotic treatment of acute appendicitis along with surgical intervention is an essential part of treatment, with a proven reduction in the incidence of early and late postoperative complications [2,3].

After acute appendicitis is diagnosed, most patients undergo surgery [1,4,5]. The clinical diagnosis is confirmed radiologically (ultrasound, computerized tomography, CT scan). There are no international guidelines on the antibiotics used alongside surgical intervention, but hospitals mostly use amoxicillin/clavulanate, piperacillin/tazobactam, third generation cephalosporins with metronidazole, carbapenems, or aminoglycosides with metronidazole in the case of a mild infection. In severe cases or high-risk patients, some guidelines indicate avoidance of aminoglycosides due to their side-effects and possible lower efficacy. Piperacillin and tazobactam in combination, carbapenems of higher generations, or cephalosporines with metronidazole (which can be substituted by clindamycin or tetracycline) are used. The empirical antibiotic therapy is based on epidemiological information, patient status, and history of other medicine used. In the case of isolation of bacteria, which is rarely performed in patients with appendicitis, the therapy may be adapted [6,7,8,9,10].

Aminoglycosides are antibiotics that are effective against Enterobacteriaceae that commonly contribute to infection in appendicitis [6]. Gentamicin was introduced in 1963 and was at that time used in Gram-negative bacteremia [11]. Later studies showed that the aminoglycosides as monotherapy could be safely used in urinary tract infections; the efficacy of aminoglycoside monotherapy in other types of infections seemed less convincing because of higher bacteriological failures [12]. Another reason that aminoglycosides are used less nowadays is their ototoxicity and nephrotoxicity. The most recent guidelines for intra-abdominal infections (IAIs) therefore recommend other antibiotics. At the same time, cephalosporins, fluoroquinolones, and carbapenems, which are currently recommended for the treatment of IAIs, are recognized as important drivers of antimicrobial resistance [10], thus contributing to antimicrobial resistance, which is a major global public health problem. In many settings the Gram-negative bacteria causing IAIs have become resistant to several recommended antibiotics, precluding their successful use [2,6]. Aminoglycosides have a structure that makes it harder for bacteria to develop resistance. The binding site of aminoglycosides is in rRNA and for bacteria to be resistant, lots of genes need to mutate, so the probability for it to happen is very low [13]. The studies on aminoglycosides used in IAIs have been performed with a multiple daily dosing regimen [12,14]. Once-daily dosing, which is widely used nowadays, is supposed to be more effective and less toxic [15,16,17] but its efficacy has not been compared to other antibiotics in IAIs. The European Committee of Antimicrobial Susceptibility Testing (EUCAST) recommends the use of aminoglycosides in extra-urinary infections together with other effective treatments including surgery. Although higher doses of aminoglycosides are recommended, it is not clear what dose is appropriate if the patient receives other effective treatment [18].

The growing problem of antimicrobial resistance coupled with the lack of new antibiotics calls for responsible use of antibiotics, including old ones, which are in many cases poorly studied. Additional studies are needed to elucidate their role in the era of antimicrobial resistance and the safe use of antimicrobials in daily practice.

Hence, we conducted a prospective randomized open-label study to compare the efficacy of a once-daily dose of GTM+MZ to two other well-recognized antibiotic treatments, ETP and CXM+MZ.

## 2. Results

### 2.1. Study Population

**Figure 1:**
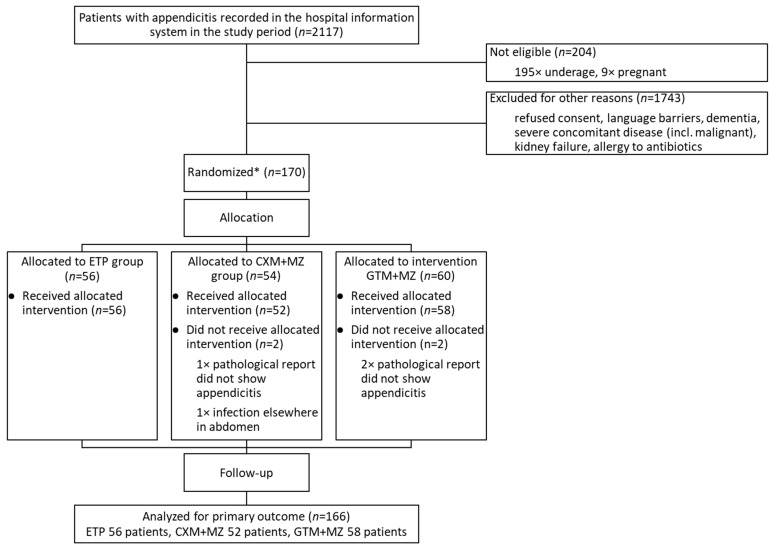
Flow diagram of progress through the phases of the randomized trial. * Screening log data were not collected; therefore, the exact number of patients assessed for eligibility and excluded prior to randomization is unavailable.

During this study, which was performed from November 2020 to April 2024, 2117 patients diagnosed with acute appendicitis were admitted to the University Medical Clinical Centre in Ljubljana (UMCL). Among them, 170 patients met the inclusion criteria and signed informed consent. Four patients were excluded, three based on their pathology reports; one of them had an appendicolith, one had a neoplasm, and one had hyperplasia of lymphatic tissue. One patient had already been operated because of appendicitis and was admitted again just for the removal of residue. ETP was prescribed to 56 patients, CXM+MZ to 52, and GTM+MZ to 58 patients (Figure 1).

There were 85 (51%) male and 81 (49%) female patients included in this study. Their mean age was 41 years with an 18–82 years range. The average body mass index (BMI) was 25.7 (SD 18.9) kg/m^2^. Appendicitis was clinically presented with nausea/vomiting in 66% of the patients, anorexia was present in 37%, 95% had pain in the right lower quadrant at home, and 17% had a fever. Total of 34% suffered from other chronic diseases. At admission, 15% had a fever above 37.5 °C, and 97% had pain in right lower quadrant of their abdomen.

Histopathology reports confirmed the diagnoses [5,8]. There were 41 patients with acute appendicitis without peritonitis, 16 with acute phlegmonous appendicitis without peritonitis, 105 with acute phlegmonous appendicitis with peritonitis, and 3 patients had lymphoid hyperplasia. There were two patients with no conclusive report (one in the CXM+MZ group and one in the GTM+MZ group).

The patients’ characteristics are summarized in Table 1, and their clinical presentations and histopathology findings are in Table 2. As expected because of randomization, there were no statistically significant differences between the groups (with all *p* > 0.2 even without an adjustment for multiple comparisons).

### 2.2. Efficacy of Antibiotic Therapy

#### 2.2.1. Duration of Treatment and Hospitalization

**Figure 2:**
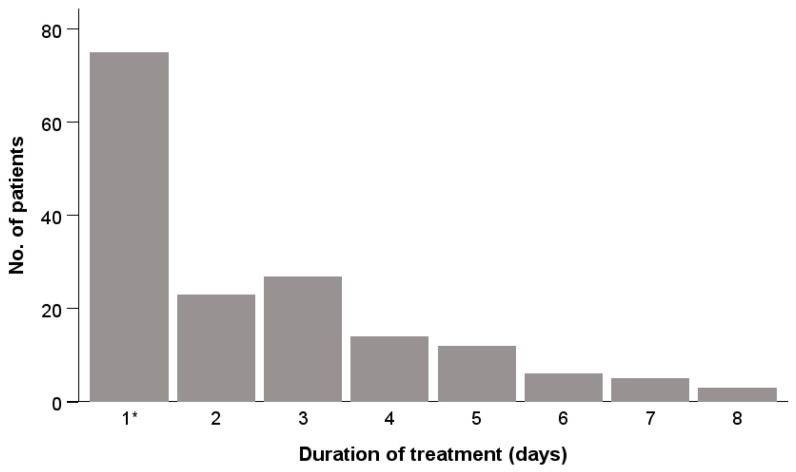
Duration of treatment in the whole sample. * This including five patients who received only one dose of CXM+MZ.

The duration of treatment is presented in Figure 2 for the whole sample. The majority, 77 patients, received an antibiotic perioperatively only, by single dose (shown in the histogram as 1 day); a total of 23 received two doses, 27 received three, and 14 received five; the others were given antibiotics for 5 days or more.

The average duration of treatment was 2.2 (SD 1.5) days in the ETP group, 2.4 (SD 1.9) days in the CXM+MZ group, and 2.9 (SD 2.0) days in the GM+MZ group. No statistical test showed a significant difference between the groups (Table 3).

The average duration of hospitalization was 3.4 (SD 1.6) days in the ETP group, 3.8. (SD 1.7) days in the CXM+MZ group, and 4.3 (SD 3.3) days in the GM+MZ group. There was no statistically significant difference between the groups (Table 3).

#### 2.2.2. Complications

The complications in all three study arms are presented in Table 4. Overall, there were only five complications during hospitalization, two of them were infectious; one patient had a psoas abscess, treated with drainage, and the other one’s inflammatory markers remained elevated, so their antibiotic therapy was changed. Four complications were registered in a month after surgery, two of them were infectious. Both patients had a superficial surgical site infection. During hospitalization, there was a borderline statistical difference in the complication rate between groups (*p* = 0.049), but there was no significant difference in the rate of infectious complications (*p* = 0.330). Within a month after surgery, there were no statistically significant differences between groups either in the rate of any complications or in the rate of infectious complications (*p* = 1.000 and *p* = 0.763, respectively).

#### 2.2.3. CRP Value

**Figure 3:**
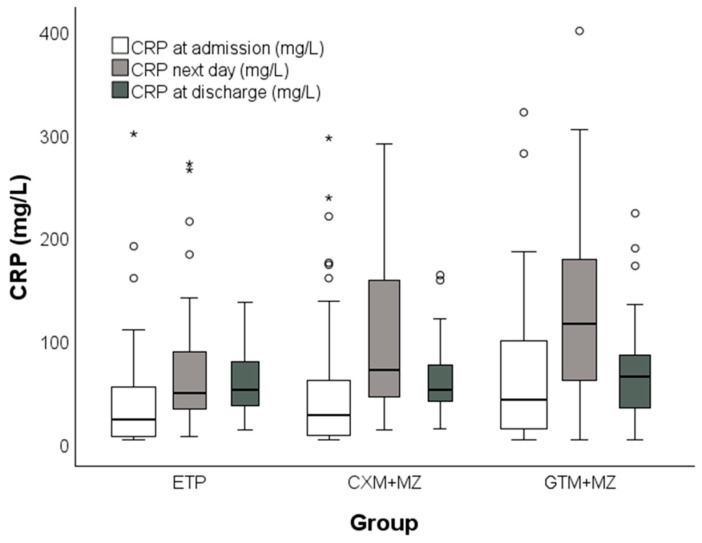
Distribution of CRP values. Thick line denotes median, box denotes interquartile range, whiskers denote non-outlier range, circles denote outliers, asterisks denote extremes.

**Figure 4:**
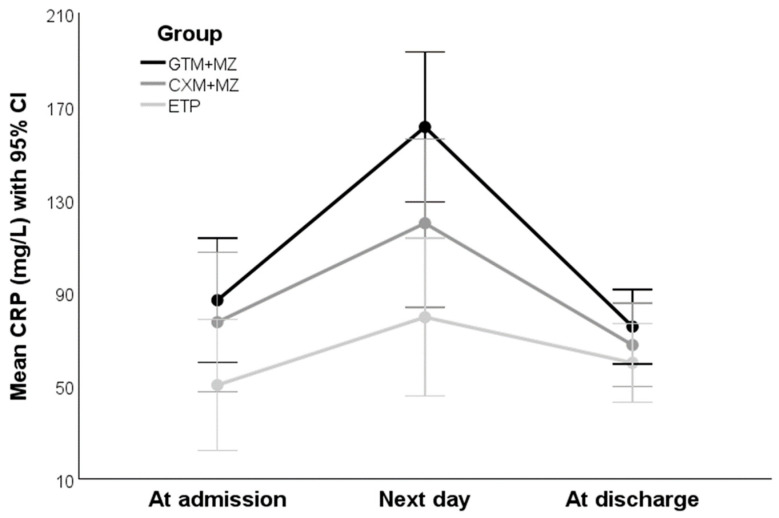
Mean values of CRP (with 95% confidence interval) for each day in each group.

The distributions of CRP are depicted with boxplots in Figure 3. The vertical axis is logarithmic with base 10, after which the distributions did not statistically significantly deviate from normality: *p* = 0.056, *p* = 0.662, and *p* = 0.088, respectively. The data were missing from 4% of patients at admission, from 30% the next day, and from 41% of patients at discharge. The data from 78 patients with complete data were used for ANOVA (26 from the ETP group, 23 from the CXM+MZ group, and 29 from the GTM+MZ group). The model assumptions were met (Box’s test of equality of covariance matrices: *p* = 0.352; Mauchly’s test of sphericity: *p* = 0.297; Levene’s test of equality of error variances based on the trimmed mean: *p* = 0.247, *p* = 0.695, and *p* = 0.304 at admission, the next day, and at discharge, respectively). ANOVA indicated that the overall CRP levels across groups differed statistically significantly between time-points (*p* < 0.001 for time effect), where an inverted U-shaped trend was observed (*p* = 0.676 for linear contrasts, *p* < 0.001 for quadratic contrasts). The overall CRP level across time-points differed statistically significantly between groups (*p* = 0.010 for group effect), but the time-trend did not differ statistically significantly between groups (*p* = 0.115 for time × group interaction). The estimated means (with error bars denoting a 95% confidence interval, CI) for the 78 complete cases are depicted in Figure 4.

The LMM accounted for about one third of the observed variance (conditional pseudo-*R*^2^ = 0.36). There was evident within-patient dependence of the CRP measurements (conditional intraclass correlation = 0.32), so fitting a LMM was justified. Like ANOVA, the LMM confirmed the statistically significant difference in the overall CRP levels between groups (*p* = 0.014) and an inverted U-shaped quadratic time-trend (*p* < 0.001), while there was no statistically significant difference in the time-course between groups (*p* = 0.122 for group × time^2^ interaction). The ETP and the CXM+MZ groups exhibited a lower overall CRP level than the GTM+MZ group (the estimated fixed-effect regression coefficients were negative: *b* = −0.32 and *b* = −0.14, respectively), but only the level of the ETP group was statistically significantly lower with respect to the GTM+MZ group (*p* = 0.003; *p* = 0.193 for CXM+MZ group).

### 2.3. Safety of Antibiotical Therapy

**Figure 5:**
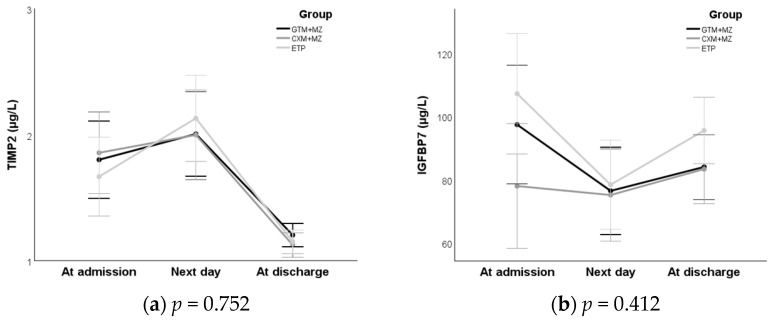

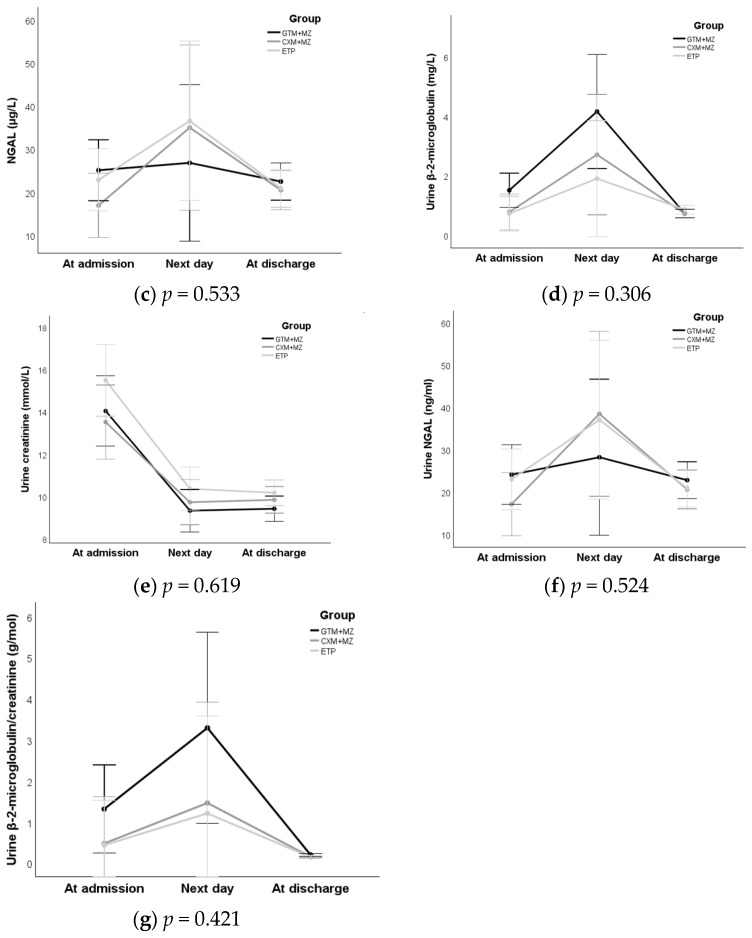
Time course of urine concentrations of biomarkers indicative of kidney injury. (**a**) Tissue inhibitor of metalloproteinases 2 (TIMP2); (**b**) insulin-like growth factor-binding protein 7 (IGFBP7); (**c**) neutrophil gelatinase-associated lipocalin (NGAL); (**d**) urine β-2-microglobulin (B2M)/creatinine; (**e**) urine creatinine; (**f**) urine NGAL; and (**g**) urine B2M. *p*-values are for the outcome × group interaction, which, if statistically significant, would be the difference in time-courses between groups.

There was no statistically significant difference between the groups either in time-trend (all *p* > 0.3) or in the overall level of the average urine biomarker values (all *p* > 0.1); for all urine biomarkers, the average values changed statistically significantly over time with a quadratic trend (all *p* < 0.03; details omitted; Figure 5).

## 3. Discussion

In this prospective randomized controlled study, we compared gentamicin in fixed once-daily doses in combination with metronidazole to two other established treatment options in patients with acute appendicitis and adequate source control. One hundred-sixty-six patients were randomized into three groups: 58 received GTM+MZ, 56 were treated with ETP, and 52 received CXM+MZ. The patients were diagnosed with acute appendicitis that presented as pain in the right lower quadrant of the abdomen. Two thirds reported nausea and approximately one third reported a loss of appetite. A higher body temperature, above 38 °C, was the least frequent sign (17%). One third of the patients suffered from chronic diseases. When admitted to the hospital, 97% still suffered from abdominal pain and 14% had body temperature above 37.5 °C at admission. Similarly, the most common sign in other studies in adults was pain in the right lower quadrant of the abdomen [5,19].

No difference was found in the average length of antibiotic therapy, or the length of hospital stay between the treatment groups. The mean treatment time was 2.5 days, while the mean hospitalization time was a day longer, 3.8 days. Some participants in other studies were given antibiotics for a longer time, from 3 to 21 days [20,21,22,23,24,25,26,27,28,29,30,31,32,33,34,35,36,37]. The longest reported treatment lasted for 49 days, with a median length of 14 days [38]. The CRP values increased to their maximum on the second day after surgical treatment and began to decline later, thus showing an inverted U-shaped-curve dynamic. Overall, all three therapies were equally efficient in lowering CRP concentration. The only statistically significant difference was between the ETP group showing the lowest increase and the other two groups. An increase of CRP could be seen in other situations related to abdominal surgery; Kerin Povsic et al. found a similar trend in patients after colorectal cancer surgery [39].

The number of complications after surgery in our study was low. Overall, only 10 patients (6%) developed any type of complications during their hospital stay and within a month after surgery. Four complications were classified as infectious. The reported rate of complications after an appendectomy in other studies ranged from 10 to 25% in the Hanberger study [40] and from 31 to 42% in an older study by Al Aloul [38]. A comparison of the rate of complications between the treatment groups in our study showed a barely significantly (*p* = 0.049) higher rate of total complications during hospital stay in the GTM+MZ group, but infectious complications did not show a statistically significant difference. Other studies comparing aminoglycosides to other antibiotics in patients with IAIs gave heterogeneous results. The studies comparing the efficacy of aminoglycosides with other drugs in acute appendicitis or other IAIs in adults are all rather old. The study by Guerra et al. from 1985 found imipenem to be the preferred treatment [41]. The efficacy was the same, but aminoglycosides were associated with more adverse effects, especially nephrotoxicity and ototoxicity. In 1982, Berne et al. compared gentamicin with clindamycin to cefamandole, and the results showed the better efficacy of aminoglycoside (*p* < 0.02) [22]. On the other hand, Jauregui et al. found cefoperazone with sulbactam more efficient than gentamicin in combination with clindamycin (*p* < 0.006) [26]. Several studies comparing aminoglycosides to carbapenems, cephalosporines, or *β*-Lactam antibiotics mainly found no statistically significant difference in efficacy [21,24,28,29,35,42,43,44].

There are no recent studies comparing the efficacy of treatment with aminoglycosides in adults; the most recent study was published in 1997 [32]. More recent articles published from 2010 onwards only reported studies on pediatric patients. Comparing a carbapenem to aminoglycoside, Dalgic et al. found no significant difference although the study showed higher rate of infectious complications in the aminoglycoside arm (*p* > 0.05) [45]. A recent study from Pogorelic showed no significant difference between ETP and GTM+MZ [46].

In the abovementioned studies in adults, aminoglycosides were used in a multiple-daily dosing regimen [20,21,22,25,26,27,28,29,30,31,32,33,34,35,36,37,42,47], up to three times a day. Once-daily dosing was only used as a dose adjustment in patients who originally received multiple daily doses [23,24,43,44,48]. An older study from 1988 suggested better efficacy if aminoglycosides were given multiple times per day [49]. Later it was shown that once-daily application is better and safer [15,22,50]. The comparable efficacy of gentamicin in our study could be related to a once-daily regimen. In addition, older studies were performed before the global increase of bacterial antimicrobial resistance that may preclude an effective use of cephalosporins or fluoroquinolones [51,52].

The main reason for discouraging the use of aminoglycosides is their toxicity; they are especially known to be nephro- and ototoxic [3,15,39,53]. In our study, we focused on nephrotoxicity that develops earlier than ototoxicity and may be expected already in short courses of gentamicin [54]. An examination of the biomarkers’ (TIMP 2, IGFBP7, NGAL, urine B2M/creatinine, urine creatinine, urine NGAL, and urine B2M) levels over time showed similar curves in all three groups of patients. Other studies showed that some biomarkers, such as NGAL, can also be elevated due to infection [55,56,57]. Following an initial increase after the first dose of antibiotic, our study showed the levels of biomarkers returning to a normal value. We also checked the creatinine, urea, and calculated glomerular filtration rates, whereby no worsening of kidney function was observed in any of the groups. A study by McWilliam showed that the level of NGAL eventuallyreturns to baseline even in cases of acute kidney injury (AKI) [58]. Other studies found that the biomarkers analyzed in our study are good, fast predictors of AKI [56,59,60,61], but on the downside they are not specific and the studies are mostly performed with rats, children, or patients that are severely ill [58,61,62,63]. Schentag et al. also examined some biomarkers in their study, B2M and alanine aminopeptidase, but their abnormalities were less frequent than in creatinine for showing renal tubular damage and nephrotoxicity [35].

The absence of nephrotoxicity in our study could be related to short treatment courses and the lower nephrotoxicity of a single-dose regimen compared to multiple-daily dosing that was used in older studies. Aminoglycosides in a single daily dose were reported to be safer than in a multiple-dosing regimen [14,17]. An older study by Prins et al. resulted in 5% renal impairment in patients receiving once-daily aminoglycoside and 24% in patients receiving it three times daily (*p* = 0.016). In that study, the patients were treated longer—for up to 16 days with a mean of 7 days (aminoglycosides administered once daily) or 7.4 days (multiple times per day) [64]. Nephrotoxicity was reported in several studies and ranged from 0 to 22%, but only a few studies have shown a trend towards the toxicity of aminoglycosides when compared to other antibiotics, and it was not statistically significant [33,34,35]. Nephrotoxicity is related to the duration of aminoglycoside therapy, but it was reported also after a single dose [65]. Two recent observational studies did not show any difference in nephrotoxicity in patients who received a short course of aminoglycosides compared to other antibiotic therapies [66,67]. Overall, nephrotoxicity appeared rarely when treating appendicitis, but the studies were mostly performed on a small number of patients and assessed children [43,68].

A relatively low dose of aminoglycosides was used in our study. EUCAST recommends 6 to 7 mg/kg body mass, while approximately 3 mg/kg lean body mass of gentamicin was used in our study. EUCAST was not able to support lower dosing because of a lack of data. All the studies referenced in the EUCAST Guidance document, including non-urinary tract infection patients treated with gentamicin, used multiple-daily dosing. In addition, the dosing is unclear when aminoglycosides are used in combination therapy, i.e., surgical control of infections as in our patients [18]. Aminoglycoside toxicity does not seem to be related to peak serum concentration, but cumulative dose is recognized as a risk factor for nephro- and ototoxicity [54].

Our study has several limitations. Firstly, the rate of complications taken into account for the sample size estimation was 10% according to the results of previous studies. With the lower rate of complications seen in the actual study, the estimated sample size would have been larger, making the results more reliable. In addition, the study was not designed and analyzed as a non-inferiority study. The number of patients randomized represented only a limited proportion of the patients admitted to hospital for acute appendicitis and operated on. The number of individuals assessed for eligibility and excluded prior to randomization was not recorded. This study was performed mostly during the COVID-19 pandemic, which made the inclusion of patients especially difficult because of the heavy workload of medical personnel in COVID-19 wards and the scarcity of resources for other activities. Nevertheless, the clinical presentations in the patients in our study did not differ much from the reports of other authors [5,19], so we believe that our cohort reflects the population of patients with acute appendicitis in general. In addition, we realized that with several patients it was not possible to obtain a sample of urine before the first antibiotic dose. The patients were typically in pain, not sufficiently hydrated, and the surgery often started shortly after admission. Some patients with a short hospital stay also denied venipuncture and urine sampling on a daily basis. To compensate for the missing values, we used the statistical methods described above. Because our patients did not have renal insufficiency on admission (it was an exclusion criterion), we may assume that the initial values of the biomarkers were normal.

## 4. Materials and Methods

### 4.1. Study Design and Population

This prospective randomized trial was conducted from November 2020 to April 2024 at the UMCL. We included patients older than 18 years, both male and female with clinically and radiologically confirmed acute appendicitis in the Emergency Department (ED) UMCL for the Clinical Department (CD) for abdominal surgery. The exclusion criteria included patients with poly-morbidity, minors, pregnant and breast-feeding women, present kidney disease, or any treatment with immunosuppressive drugs. Before enrolment in this study, the patients signed informed consent.

### 4.2. Randomization and Treatment Allocation

We randomly allocated participants to one of three antibiotic groups. Simple randomization was computer-generated using Excel function RAND [69]. Each patient was allocated to one of the three treatment groups in a 1:1:1 ratio. The recruiters were blinded to the assignment sequence.

### 4.3. Procedures

The participants were diagnosed at the ED and randomly assigned to one of the treatment groups. They all underwent surgery (laparoscopic appendectomy). The first dose of antibiotics was given by the anesthesiologist before incision. They were given either ertapenem 1 g q24 h, gentamicin 240 mg q24 h (≈3 mg/kg) with metronidazole 500 mg q8 h, or cefuroxime 1.5 g q8 h with metronidazole 500 mg q8 h. All antibiotics were given intravenously. The relatively low dose of gentamicin is used routinely in the hospital in surgically treated patients. The practice was not changed because there are no clear EUCAST recommendations on dosing gentamicin in combination with other effective therapies [18]. Gentamicin serum concentration measurements were not performed because of the short treatment duration [65]. The duration of antibiotic therapy and length of hospital stay were set by the treating physician according to the type of inflammation seen intra-operatively and the patient’s condition.

Urine and blood samples were collected on the day of admission before the first antibiotic dose, one day after surgery and on the day of discharge if that day was different from the day after surgery, or the day thereafter (day 2 after surgery). The urine samples for urine biomarkers were immediately stored in the refrigerator at 6 °C. The samples were transferred to the Institute of Clinical Chemistry and Biochemistry (ICCB) at the UMCL.

One month after the surgery the follow up was done using the UMCL’s hospital information system, and the Central Patient Data Repository where we got the information on possible complications or later admissions to any other hospital or clinic in Slovenia. Complications were evaluated using the QC (Quality Control) 1 Form, which is available and in routine use at the Department for Abdominal Surgery, which is based on the Clavien–Dindo classification. The classification is based on the severity of complications and consists of grades. In the first grade, there are complications that do not need any therapy but there is a deviation from the normal postoperative course. Drugs such as antiemetics, antipyretics, analgetics, diuretics, electrolytes, and physiotherapy can be used. In Grade 2, any drug can be used, including parenteral nutrition or blood transfusion. Grade 3 are complications that require radiological, surgical, or endoscopic intervention either with or without general anesthesia. Grade 4 means life-threatening complication, including single or multi-organ dysfunction. Grade 5 means that the patient died. Infectious clinical examples can be found in different grades, Grade 1 involves wound infections, Grade 2 involves infectious diarrhea, a renal or urinary tract infection requiring antibiotics, and phlegmona, while in Grade 3 there are wound infections leading to eventration of small bowel, and Grade 4 includes necrotizing pancreatitis [70].

### 4.4. Laboratory Methods

Urine and blood samples were transported to ICCB. Blood samples were centrifuged at 1500× *g* for 10 min and the serum was separated from the clotted blood. All urine and serum samples were frozen and stored at −20 °C until analysis.

All urine samples were analyzed in one batch. The concentration of NGAL in urine was measured using a two-step luminescent immunoassay with intra-laboratory CV < 5% and the limit of quantification was 10 µg/L (Architect analyzer, Abbott Diagnostics, Lake Forest, CA, USA). For the measurement of IGFBP7 in urine, sandwich ELISA immunoassays with a limit of detection 0.03 µg/L were used (BioVendor, Brno, Czech Republic). The same principle of the ELISA immunoassay was used for the measurement of TIMP-2 in urine (Antibodies-online GmbH, Aachen, Germany) with a limit of detection 0.1 µg/L. The TIMP-2/IGFBP7 ratio was calculated for result interpretation.

Because the manufacturer recommends the use of fresh urine samples, we investigated the stability of B2M in frozen urine samples during a freeze–thaw cycle.

Ten fresh urine samples covering a broad B2M concentration range were collected and prepared by adding one drop of urine stabilizer per milliliter, adjusting the pH to 7–9, and centrifuging at 400× *g* for 5 min. The samples were analyzed immediately on a BN II immunonephelometer (Siemens Healthineers, Forchheim, Germany) using N Latex β-2-Microglobulin Reagent (Siemens Healthineers, Forchheim, Germany). After labeling, the samples were stored at −20 °C for two months and reanalyzed; the turbid samples were centrifuged at 15,000× *g* for 10 min prior to measurement. To assess stabilizer effects, ten additional samples were stored under identical conditions without a stabilizer, and three samples with varying creatinine concentrations were analyzed before and after the stabilizer addition to evaluate potential interference.

Analytical principle: B2M forms immune complexes with latex-bound antibodies; light scattering intensity is proportional to concentration.

Stability conclusion: Urinary B2M remained stable for at least two months under the applied storage conditions (−20 °C).

### 4.5. Outcomes and Definitions

The primary outcome was clinical responses to the treatment. We analyzed the length of treatment and hospitalization, the CRP dynamics, the total number of complications, and infectious complications during treatment in the hospital and in the month after surgery.

Safety was evaluated via kidney function using various urine biomarkers. TIMP2 (µg/L), IGFBP7 (µg/L), NGAL (µg/L), urine B2M (mg/L), urine creatinine (mmol/L), urine B2M (g/mol), and urine NGAL (µg/L) were determined.

### 4.6. Statistical Analysis

Sample size was estimated based on the preliminary results of aminoglycosides safety and efficacy studies in the literature. We assumed the statistical testing of differences between groups of equal size with an alpha error-rate of 5% and statistical power of 80% (beta error of 20%).

Frequency distributions and descriptive statistics were calculated for all the studied variables. The average duration of treatment and hospitalization were compared between groups using ANOVA, the Welch test, and the Kruskal−Wallis test to reach a reliable conclusion. Histopathology findings, clinical presentation, and rate of complications were compared between groups using the extended Fisher’s exact test. Confidence intervals for proportions were estimated using Wilson’s method. The assumption of data missing at random was verified for all the outcomes using Little’s MCAR test (*p* = 0.290).

The normal-distribution fit of CRP measurements was assessed using Kolmogorov−Smirnov test. The time-course of CRP measurements was compared between groups using two-way mixed ANOVA (which requires complete-case analysis, so only the patients with all three measurements were included; time-point was included as a within-subjects factor and group was included as a between-subjects factor) and a LMM, which used all the available data; a growth model of log-transformed measurements with a random intercept was fitted, whereby the time-point value was centered and squared to test for a quadratic trend).

For the other biomarkers, missing data were replaced using an expectation-maximization (EM) algorithm. The time-course of those biomarkers was compared between groups using two-way mixed ANOVA on the imputed data, whereby time-point was included as a within-subjects factor and group was included as a between-subjects factor. When Mauchly’s test indicated a violation of the assumption of sphericity, Greenhouse−Geisser correction was applied. If the effects of the group and interaction were not statistically significant, and the effect of time was, the overall time-trend was tested using linear and quadratic contrasts. Statistical analyses were performed using IBM SPSS Statistics 29 (IBM Corp., Armonk, NY, USA, 2023).

### 4.7. Study Design and Registration

The design of this study complies with the CONSORT Guidelines (Appendix A). This study was registered in 2020 on the ISRCTN registry with the study ID ISRCTN15780064.

## 5. Conclusions

Our randomized open-label study did not show clinically relevant differences of once-daily dosing of a relatively low dose of gentamicin combined with metronidazole in comparison to two other well-established therapeutic regimens with beta-lactam antibiotics in patients with immediately performed source control for acute appendicitis. The observed efficacy and safety were comparable. Using short courses of gentamicin instead of broad-spectrum beta-lactams may be effective because of potential antimicrobial resistance in enteric pathogens in some settings. At the same time, gentamicin has a potential to be used as a carbapenem- and cephalosporin-saving drug, thus increasing the diversity of antibiotic use and decreasing selective pressure related to the use of other antimicrobials. We hope that the results of our study will stimulate further research of the use of old antibiotics including aminoglycosides.

## Figures and Tables

**Table 1:** Patients’ characteristics.

	Total (*n* = 166)	ETP (*n* = 56)	CXM+MZ (*n* = 52)	GTM+MZ (*n* = 58)
Age Mean (median, range)	42 (41, 18–82)	41 (37, 18–82)	42 (39, 18–81)	44 (44, 19–81)
BMI	(*n* = 127)	(*n* = 42)	(*n* = 41)	(*n* = 44)
Mean (median, range)	25.7 (25.0, 18.0–44.6)	25.0 (24.6, 18.0–35.3)	26.3 (24.5, 18.4–44.6)	25.8 (25.6, 18.1–35.8)
Sex no. (%) male	85 (51)	30	28	27
female	81 (49)	26	24	31

**Table 2:** Clinical presentation histopathology results in patients with acute appendicitis.

	Total No. (%)	ETP	CXM+MZ	GTM+MZ
Nausea at home	108 (66)	38	31	39
Loss of appetite	62 (37)	20	19	23
History of pain in right lower quadrant	158 (95)	54	50	54
Chronic disease	56 (34)	18	15	23
Fever at home > 38 °C	27 (17)	7	6	14
High body temperature at admission	24 (14)	6	7	11
Pain in right lower quadrant at admission	161 (97)	56	49	56
Histopathology report				
acute appendicitis	41	17	11	12
phlegmonous appendicitis	16	4	6	6
phlegmonous appendicitis with peritonitis	105	33	33	39
lymphoid hyperplasia	3	2	1	
Total	166	56	52 *	58

* one sample was destroyed during transport and histopathology was no longer possible.

**Table 3:** Descriptive statistics and results of statistical tests for comparing duration of treatment and length of hospital stay between the three treatment groups.

		Whole Sample	ETP	CXM+MZ	GTM+MZ	*p* (ANOVA)	*p* (Welch)	*p* (Kruskal-Wallis)
Duration of treatment (days)	Mean (SD)	2.5 (1.8)	2.2 (1.5)	2.4 (1.9)	2.9 (2.0)	0.090	0.093	0.084
	Median (range)	2 (1–8)	2 (1–7)	1 (1–8)	3 (1–8)			
Length of hospital stay (days)	Mean (SD)	3.8 (2.4)	3.4 (1.6)	3.8 (1.7)	4.3 (3.3)	0.192	0.222	0.451
	Median (range)	3 (2–18)	3 (2–9)	3 (2–9)	3 (2–18)			

**Table 4:** Complications and infectious complications during hospitalization and within one month after surgery.

	ETP (*n* = 56)	CXM+MZ (*n* = 52)	GTM+MZ (*n* = 58)	Total (*n* = 166)
**Complications during hospitalization (*p* = 0.049)**				
No	55 (98%)	52 (100%)	53 (91%)	160 (96%)
Clavien–Dindo class 1	1 (2%)	0 (0%)	4 (7%)	5 (3%)
Clavien–Dindo class 2	0 (0%)	0 (0%)	1 (2%)	1 (1%)
Proportion [95% confidence interval]	1.8% (0.3–9.5%)	0.0% (0.0–6.9%)	8.6% (3.7–18.6%)	3.6% (1.7–7.7%)
**Infectious complications during hospitalization (*p* = 0.330)**				
No	56 (100%)	52 (100%)	56 (97%)	164 (99%)
Yes	0 (0%)	0 (0%)	2 (3%)	2 (1%)
Proportion [95% confidence interval]	0.0% (0.0–6.4%)	0.0% (0.0–6.9%)	3.6% (1.0–12.1%)	1.2% (0.3–4.3%)
**Complications within a month after surgery (*p* = 1.000)**				
No	55 (98%)	51 (98%)	56 (97%)	162 (98%)
Clavien–Dindo class 1	1 (2%)	0 (0%)	1 (2%)	2 (1%)
Clavien–Dindo class 2	0 (0%)	1 (2%)	1 (2%)	2 (1%)
Proportion [95% confidence interval]	1.8% (0.3–9.5%)	1.9% (0.3–10.1%)	3.4% (1.0–11.7%)	2.4% (0.9–6.0%)
**Infectious complications within a month after surgery (*p* = 0.763)**				
No	56 (100%)	51 (98%)	57 (98%)	164 (99%)
Yes	0 (0%)	1 (2%)	1 (2%)	2 (1%)
Proportion [95% confidence interval]	0.0% (0.0–6.4%)	1.9% (0.3–10.1%)	1.7% (0.3–9.1%)	1.2% (0.3–4.3%)

## Data Availability

Data are available to interested researchers upon request.

## Abbreviations

The following abbreviations are used in this manuscript:

GTM+MZ	Gentamicin with metronidazole
ETP	Ertapenem
CXM+MZ	Cefuroxime with metronidazole
CRP	C-reactive protein
ANOVA	Analysis of variance
LMM	Linear mixed model
AAST	American Association for the surgery of Trauma
IAIs	Intra-abdominal infections
EUCAST	European Committee of Antimicrobial Susceptibility Testing
UMCL	University Medical Clinical Centre in Ljubljana
BMI	Body mass index
TIMP2	Tissue inhibitor of metalloproteinases 2
IGFBP7	Insulin-like growth factor-binding protein 7
NGAL	Neutrophil gelatinase-associated lipocalin
B2M	β-2-Microglobulin
AKI	Acute kidney injury
ED	Emergency Department
CD	Clinical Department
ICCB	Institute of Clinical Chemistry and Biochemistry
EM	Expectation-maximization

## Appendix A

**Table A1:** CONSORT 2025 checklist [71].

Section/Topic	No.	CONSORT 2025 Checklist Item Description	Reported on Page No.
**Title and abstract**			
Title and structured abstract	1a	Identification as a randomized trial. Described in the Section 4.	Page 12
	1b	Structured summary of the trial design, methods, results, and conclusions.	Page 1
**Open science**			
Trial registration	2	Name of trial registry, identifying number (with URL), and date of registration. Described in the Section 4. Study is listed on the ISRCTN registry with the study ID ISRCTN15780064. https://www.isrctn.com/search?q=ISRCTN15780064 (accessed on 29 October 2020).	Page 14
Protocol and statistical analysis plan	3	Where the trial protocol and statistical analysis plan can be accessed. It can be accessed in the Section 4. The details are available from the authors, on request, in the Slovenian language.	Pages 12 to 14
Data sharing	4	Where and how the individual de-identified participant data (including data dictionary), statistical code, and any other materials can be accessed. The data are available from the authors, on request, in the Slovenian language.	Page 15
Funding and conflicts of interest	5a	Sources of funding and other support (e.g., supply of drugs), and the role of funders in the design, conduct, analysis, and reporting of the trial. This study was partly funded by University Medical Centre Ljubljana Research Grants (20200024 and 20240054). The drugs are regularly used in hospital for the study indication. There was no influence of funding on any part of this study,	Page 16
	5b	Financial and other conflicts of interest of the manuscript authors. No conflicts of interest.	Page 16
**Introduction**			
Background and rationale	6	Scientific background and rationale.	Page 2 to 4
Objectives	7	Specific objectives related to benefits and harms.	Page 15
**Methods**			
Patient and public involvement	8	Details of patient or public involvement in the design, conduct, and reporting of the trial. No patient or public involvement in the design; all patients were informed about the study and signed an informed consent form.	Page 12
Trial design	9	Description of trial design including type of trial (e.g., parallel group, crossover), allocation ratio, and framework (e.g., superiority, equivalence, non-inferiority, exploratory).	Page 12 to 15
Changes to trial protocol	10	Important changes to the trial after it commenced including any outcomes or analyses that were not pre-specified, with reason. No important changes in the design.	-
Trial setting	11	Settings (e.g., community, hospital) and locations (e.g., countries, sites) where the trial was conducted.	Pages 12 and 13
Eligibility criteria	12a	Eligibility criteria for participants.	Page 12
	12b	If applicable, eligibility criteria for sites and for individuals delivering the interventions (e.g., surgeons, physiotherapists).	Not applicable
Intervention and comparator	13	Intervention and comparator with sufficient details to allow replication. If relevant, it is where additional materials describing the intervention and comparator (e.g., intervention manual) can be accessed. No additional materials.	Pages 12 to 15
Outcomes	14	Pre-specified primary and secondary outcomes, including the specific measurement variable (e.g., systolic blood pressure), analysis metric (e.g., change from baseline, final value, time to event), method of aggregation (e.g., median, proportion), and time-point for each outcome.	Pages 14 and 15
Harms	15	How harms were defined and assessed (e.g., systematically, non-systematically).	Page 13 and 14
Sample size	16a	How sample size was determined, including all assumptions supporting the sample size calculation.	Page 14
	16b	Explanation of any interim analyses and stopping guidelines. No interim analysis.	-
Randomization:			
Sequence generation	17a	Who generated the random allocation sequence and the method used. Randomizing was made with Excel. We had 3 groups and 3 different antibiotics to determine. Statistics specified the number of subjects.	Page 13
	17b	Type of randomisation and details of any restriction. Simple randomization using Excel—the function RAND. We randomized patients before starting the study so everyone who had been included was prescribed a specific antibiotic. Some of the patients were not meeting the including criteria and that is why there is a different number of patients in each study arm.	Page 13
Allocation concealment mechanism	18	Mechanism used to implement the random allocation sequence (e.g., central computer/telephone; sequentially numbered, opaque, sealed containers), describing any steps to conceal the sequence until interventions were assigned. We used a computer software program (Excel) that generates the random sequence. Random antibiotics were given to each subject. Paper medical records with already assigned treatments were prepared by investigators (NO) in advance in closed envelopes and the doctors in the ED used them sequentially. The recruiters were blinded to the assignment sequence.	Page 13
Implementation	19	Whether the personnel who enrolled and those who assigned participants to the interventions had access to the random allocation sequence. The personnel who enrolled the patients had access to the random allocation sequence.	
Blinding	20a	Who was blinded after assignment to interventions (e.g., participants, care providers, outcome assessors, data analysts). It was an open-label study.	Page 1, page 12.
	20b	If blinded, how blinding was achieved and a description of the similarity of interventions.	-
Statistical methods	21a	Statistical methods used to compare groups for primary and secondary outcomes, including harms.	Pages 14 and 15
	21b	Definition of who is included in each analysis (e.g., all randomized participants), and in which group.	Pages 5 to 8
	21c	How missing data were handled in the analysis.	Pages 14 and 15
	21d	Methods for any additional analyses (e.g., subgroup and sensitivity analyses), distinguishing pre-specified from post hoc. Not performed.	-
**Results**			
Participant flow, including flow diagram	22a	For each group, the numbers of participants who were randomly assigned, received intended intervention, and were analysed for the primary outcome.	Pages 4 and 5
	22b	For each group, losses and exclusions after randomisation, together with reasons.	Page 4
Recruitment	23a	Dates defining the periods of recruitment and follow-up for outcomes of benefits and harms.	Page 14
	23b	If relevant, why the trial ended or was stopped.	Not relevant
Intervention and comparator delivery	24a	Intervention and comparator as they were actually administered (e.g., where appropriate, who delivered the intervention/comparator, how participants adhered, whether they were delivered as intended [fidelity]). All antibiotic therapy was given intravenously and recorded in medical records per protocol of the routine care.	-
	24b	Concomitant care received during the trial for each group. The patients received routine care.	-
Baseline data	25	A table showing baseline demographic and clinical characteristics for each group.	Page 5
Numbers analysed, outcomes and estimation	26	For each primary and secondary outcome, by group: The number of participants included in the analysis. The number of participants with available data at the outcome time-point. Result for each group, and the estimated effect size and its precision (such as 95% confidence interval). For binary outcomes, presentation of both absolute and relative effect size.	Pages 4 to 10
Harms	27	All harms or unintended events in each group.	Pages 7 to 10
Ancillary analyses	28	Any other analyses performed, including subgroup and sensitivity analyses, distinguishing pre-specified from post hoc. No other analysis.	-
**Discussion**			
Interpretation	29	Interpretation consistent with results, balancing benefits and harms, and considering other relevant evidence.	Pages 10 to 12
Limitations	30	Trial limitations, addressing sources of potential bias, imprecision, generalisability, and, if relevant, multiplicity of analyses.	Pages 12 and 13
